# Time trend of axial length and associated factors in 4- and 5-year-old children in Shanghai from 2013 to 2019

**DOI:** 10.1007/s10792-020-01637-5

**Published:** 2020-11-12

**Authors:** Tao Li, Ting Wan, Xiaoqian Yao, Huihong Qi, Xuefeng Chen, Man She, Qianqian Hu, Xiaodong Zhou

**Affiliations:** 1 Department of Ophthalmology, Jinshan Hospital of Fudan University, 1508 Longhang Road, Shanghai, 201508 China; 2 Department of Ophthalmology, Eye and ENT Hospital of Fudan University, Shanghai, China; 3 Center of Eye Disease Prevention, Jinshan District, Shanghai, China

**Keywords:** Axial length, Preschool-age children, Time trend, Associated factors

## Abstract

**Purpose:**

To evaluate the time trend of axial length (AL) and associated factors in 4- and 5-year-old children in Shanghai from 2013 to 2019.

**Methods:**

This was a 7-year observational study of 985 four-year-old and 1059 five-year-old children in Shanghai. AL, horizontal and vertical corneal curvature, spherical equivalent (SE), and body height and weight were measured. Furthermore, a questionnaire was collected, including time outdoors and bad eyesight habits.

**Results:**

In 4-year-old children, no significant difference was found in AL (*P* = 0.526), but significant differences were observed in SE (*P* = 0.001), horizontal corneal curvature (*P* = 0.006), vertical corneal curvature (*P* = 0.004), height (*P* < 0.001), and weight (*P* = 0.022) from 2013 to 2019. In 5-year-old children, no significant differences were found in AL (*P* = 0.304), SE (*P* = 0.200), or weight (*P* = 0.292), but significant differences were observed in horizontal corneal curvature (*P* = 0.040), vertical corneal curvature (*P* = 0.015), and height (*P* < 0.001) from 2013 to 2019. Multivariate analyses revealed that AL was mainly significantly associated with boys and time outdoors in the 4- and 5-year-old children.

**Conclusions:**

The AL of 4- and 5-year-old children remained relatively stable in Shanghai from 2013 to 2019. Longitudinal studies are needed to confirm the relationship between AL elongation and environmental risk factors.

## Introduction

Myopia, which is the result of a mismatch between the axial length (AL) and ocular refractive power, has become a major public health issue worldwide [1]. AL is thus an important anatomic parameter for the optics of the eye, determining the refraction. Furthermore, the AL may be the primary factor for the eventual development of myopia-related visual impairment complications, such as retinal detachment, optic disc abnormalities, myopic macular degeneration, and choroidal neovascularization [2–4]. The risk of visual impairment is highest for those with high myopia, especially when having an AL above 30 mm [5]. Therefore, AL may be a principal determinant of ocular function.

Vision screening is recommended at least once in all children aged 3–5 years old by the US Preventive Services Task Force [6]. Despite the importance of AL, few population-based studies have focused on AL, especially in preschool children [7–12]. The purpose of this study is to evaluate the time trend of AL in 4- and 5-year-old children, and to assess potential factors associated with AL in these children in a kindergarten in Shanghai from 2013 to 2019.

## Methods

### Subjects

This was a 7-year observational study in Jinshan District in the southwest of Shanghai, China. Children aged 4 or 5 years from one kindergarten were included in the study, including 985 four-year-olds and 1059 five-year-olds. The exclusion criteria were history of severe ocular diseases (e.g., cataract, glaucoma, strabismus, and amblyopia) and surgeries.

The study was approved by the Ethics Committee of Jinshan Hospital of Fudan University, China. All study procedures adhered to the tenets of the Declaration of Helsinki. Written informed consent was obtained from the parents or guardians of all children.

### Examination

The annual visit was completed between 1 September and 31 December from 2013 to 2019, except for 2015, when no ocular examination was carried out for kindergarten children. An ocular biometry system (IOL Master; Carl Zeiss Meditec, Oberkochen, Germany) was used to obtain the axial length (AL), and horizontal and vertical corneal curvature. The mean value of five good measurements was used in the analysis. According to our previous studies [13, 14], an auto-refractor (RK-F1; Canon Corporation, Tokyo, Japan) was used to measure refraction under non-cycloplegic conditions. The mean value of three good measurements was used in the analysis.

Furthermore, body height and weight were recorded for all children, measured in a standard manner without shoes or thick clothes.

In addition, a questionnaire regarding daily life activities was completed by the child’s parents or guardians when the child started school every September. Time spent outdoors was obtained using questions such as “How much time does your child spend outdoors every day?” separately for weekdays and weekend days. The mean number of hours per day was calculated as time spent during weekdays × 5/7 + time spent on weekend days × 2/7. Furthermore, dichotomous variables (coded 1 or 0) were also recorded, including gender (1 = boy, 0 = girl) and bad eyesight habits, such as continuously reading books, writing homework, or playing with electronic devices for 30–40 min (1 = none, 0 = frequent or sometimes), excessive bending, suggesting a distance between the eye and desk of less than 33 cm (1 = none, 0 = frequent or sometimes), watching TV from a distance of less than 2 m (1 = none, 0 = frequent or sometimes), and wrong sitting posture, suggesting a distance between the thorax and desk of less than the size of a fist (1 = none, 0 = frequent or sometimes).

### Statistical analysis

Both eyes of each child were examined, but only data from the right eye was used for the analysis. Spherical equivalent (SE) was calculated as spherical power + 0.5 negative cylinder power. According to previous studies [13, 15], myopia was defined as SE ≤ −1.00 D, emmetropia as −1.00 D < SE <  +2.00 D, and hyperopia as SE ≥ +2.00 D.

SPSS V.17.0 software was used for data analysis. One-way analysis of variance with the Bonferroni post hoc test was used to analyze the differences of covariates for 4- and 5-year-old children from 2013 to 2019. Independent *t*-test was used to analyze the differences of AL and SE between all the 4- and 5-year-old children. Multivariate linear regression analysis was performed to assess the potential associated factors for AL. All *p* values were two-sided and considered statistically significant when less than 0.05.

## Results

**Fig. 1:**
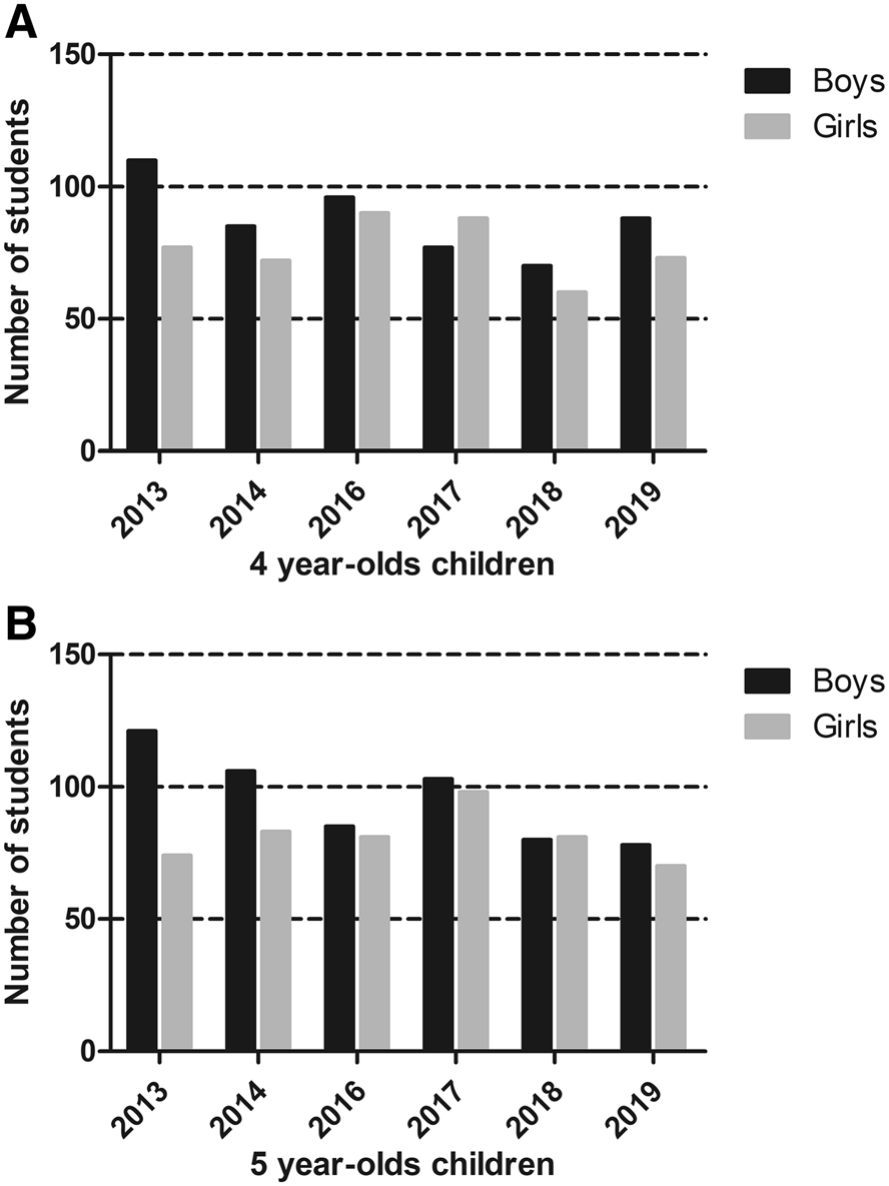
Number of 4- and 5-year-old children

As shown in Fig. 1a, for the 4-year-olds, there were 187 including 77 (41.2%) girls in 2013, 157 children including 72 (45.9%) girls in 2014, 186 children including 77 (48.4%) girls in 2016, 165 children including 88 (53.3%) girls in 2017, 129 children including 60 (46.5%) girls in 2018, and 161 children including 73 (45.3%) girls in 2019. As shown in Fig. 1b, for the 5-year-olds, there were 195 children including 74 (37.9%) girls in 2013, 188 children including 83 (44.1%) girls in 2014, 166 children including 81 (48.8%) girls in 2016, 201 children including 98 (48.8%) girls in 2017, 161 children including 81 (50.3%) girls in 2018, and 148 children including 70 (47.3%) girls in 2019.

**Fig. 2:**
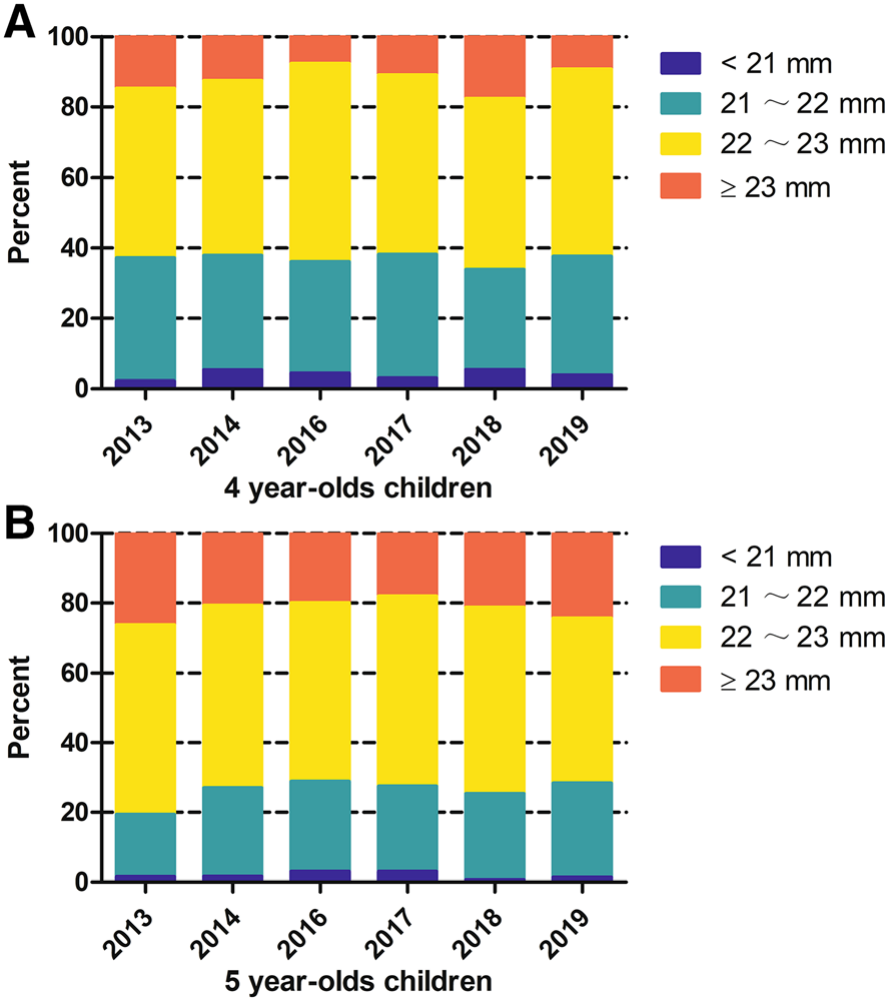
Percentages of different axial length of 4- and 5-year-old children

**Fig. 3:**
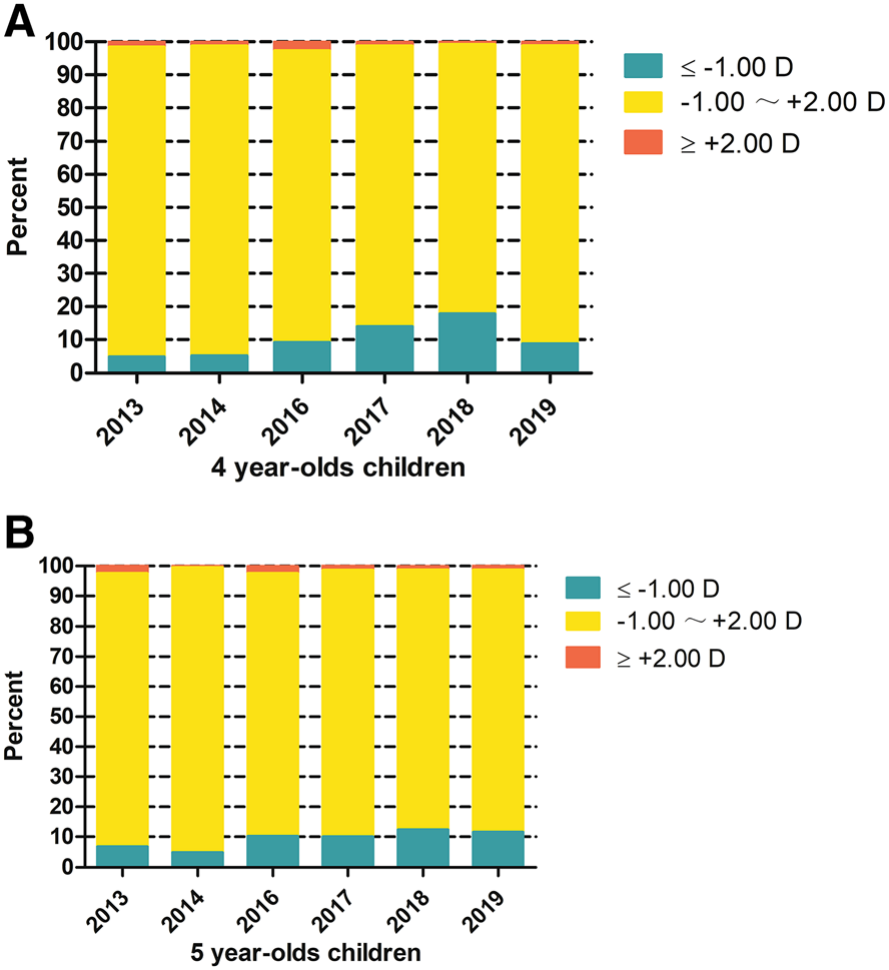
Percentages of different spherical equivalent of 4- and 5-year-old children

The mean AL of the 4- and 5-year-old children was 22.21 ± 0.69 mm and 22.45 ± 0.70 mm, respectively, with significant difference (*P* < 0.001) from 2013 to 2019. The most common AL range was 22–23 mm in the 4-year-old children (Fig. 2a; 48.1% in 2013, 49.7% in 2014, 56.3% in 2016, 50.9% in 2017, 48.5% in 2018, and 53.1% in 2019) and 5-year-old children (Fig. 2b; 54.4% in 2013, 52.4% in 2014, 51.2% in 2016, 54.5% in 2017, 53.4% in 2018, and 47.3% in 2019). The mean SE of the 4- and 5-year-old children was 0.03 ± 1.73 D and 0.06 ± 1.30 D, respectively, without significant difference (*P* = 0.700) from 2013 to 2019. The most common refraction was emmetropia in 4-year-old children (Fig. 3a; 93.6% in 2013, 93.6% in 2014, 88.2% in 2016, 84.8% in 2017, 81.4% in 2018, and 90.1% in 2019) and 5-year-old children (Fig. 3b; 90.8% in 2013, 94.7% in 2014, 87.3% in 2016, 88.6% in 2017, 86.3% in 2018, and 87.2% in 2019).

As shown in Table 1, no significant difference was found in AL (*P* = 0.526) in 4-year-old children from 2013 to 2019, whereas significant differences were observed in SE (*P* = 0.001), horizontal corneal curvature (*P* = 0.006), vertical corneal curvature (*P* = 0.004), height (*P* < 0.001), and weight (*P* = 0.022) from 2013 to 2019. Bonferroni post hoc tests further showed that SE was higher in 2013 than in 2017, 2018, and 2019 (all *P* < 0.05). Horizontal and vertical corneal curvature were smaller in 2013 than in 2016 (*P* = 0.002 and *P* = 0.002, respectively). Height was lower in 2013 than in 2014, 2017, 2018, and 2019 (all *P* < 0.05), greater in 2014 than in 2016, 2017, 2018, and 2019 (all *P* < 0.05), and lower in 2016 than in 2018 (*P* = 0.016). Although there was a significant difference in weight (*P* = 0.022) from 2013 to 2019, no significant differences were observed in paired comparisons (all *P* > 0.05). As shown in Table 2, no significant differences were found in AL (*P* = 0.304), SE (*P* = 0.200), or weight (*P* = 0.292) in 5-year-old children from 2013 to 2019, whereas significant differences were observed in horizontal corneal curvature (*P* = 0.040), vertical corneal curvature (*P* = 0.015), and height (*P* < 0.001) from 2013 to 2019. Bonferroni post hoc tests further showed that vertical corneal curvature was smaller in 2013 than in 2017 (*P* = 0.016), whereas horizontal corneal curvature was marginally smaller than in 2017 (*P* = 0.050). Height was lower in 2013 than in 2014, 2017, 2018, and 2019 (all P < 0.05), but greater in 2014 than in 2016, 2017, and 2018 (all *P* < 0.05).

**Table 1:** Demographic characteristics of 4-year-old children

	2013	2014	2016	2017	2018	2019	*P*
Axial length (mm)	22.24 ± 0.70	22.18 ± 0.72	22.16 ± 0.66	22.21 ± 0.64	22.30 ± 0.72	22.19 ± 0.72	0.526
Spherical equivalent (D)	0.49 ± 3.15	0.11 ± 0.75	−0.01 ± 1.22	−0.17 ± 1.09	−0.31 ± 1.77	−0.07 ± 1.82	0.001
Horizontal corneal curvature (D)	42.22 ± 3.78	42.71 ± 1.37	42.10 ± 6.58	42.78 ± 1.45	42.49 ± 1.62	42.75 ± 1.27	0.006
Vertical corneal curvature (D)	43.32 ± 3.91	43.84 ± 1.47	43.23 ± 6.75	43.90 ± 1.61	43.58 ± 1.78	43.96 ± 1.46	0.004
Height (cm)	105.8 ± 4.9	113.9 ± 7.5	107.2 ± 9.0	107.9 ± 4.4	109.5 ± 5.1	108.2 ± 4.9	< 0.001
Weight (kg)	18.0 ± 9.9	19.2 ± 3.9	18.4 ± 5.2	17.9 ± 2.4	19.7 ± 2.9	18.2 ± 8.2	0.022

**Table 2:** Demographic characteristics of 5-year-old children

	2013	2014	2016	2017	2018	2019	*P*
Axial length (mm)	22.51 ± 0.67	22.43 ± 0.67	22.40 ± 0.79	22.35 ± 0.70	22.46 ± 0.67	22.46 ± 0.72	0.304
Spherical equivalent (D)	0.26 ± 2.30	0.06 ± 0.66	0.03 ± 1.03	0.03 ± 0.99	0.01 ± 0.89	−0.10 ± 1.01	0.200
Horizontal corneal curvature (D)	42.17 ± 2.84	42.49 ± 3.51	42.74 ± 1.37	42.83 ± 1.42	42.76 ± 1.44	42.69 ± 1.41	0.040
Vertical corneal curvature (D)	43.29 ± 2.92	43.53 ± 3.63	43.81 ± 1.37	44.06 ± 1.53	43.81 ± 1.58	43.95 ± 1.64	0.015
Height (cm)	112.7 ± 5.4	116.9 ± 5.6	114.2 ± 5.6	114.6 ± 4.6	114.7 ± 4.8	115.4 ± 4.4	< 0.001
Weight (kg)	20.3 ± 7.9	21.2 ± 3.8	21.2 ± 5.5	21.0 ± 3.9	21.3 ± 3.1	21.4 ± 3.6	0.292

As shown in Table 3, for the 4-year-old children, multivariate analysis revealed that AL was significantly associated with boys (*P* < 0.001) in 2013, with time outdoors (*P* < 0.001) in 2014, with time outdoors (*P* < 0.001) in 2016, with boys (*P* < 0.001) in 2017, with boys (*P* < 0.001) in 2018, and with boys (*P* < 0.001) in 2019. As shown in Table 4, for 5-year-old children, multivariate analysis revealed that AL was significantly associated with boys (*P* < 0.001) and time outdoors (*P* = 0.048) in 2013, with time outdoors (*P* < 0.001) in 2014, with time outdoors (*P* < 0.001) and continuously using eyes for 30–40 min (*P* = 0.018) in 2016, with boys (*P* = 0.001) in 2017, with boys (*P* < 0.001) and time outdoors (*P* = 0.018) in 2018, and with boys (*P* = 0.023) in 2019.

**Table 3:** Multivariate linear regression analysis for potential factors associated with axial length in 4-year-old children

	2013		2014		2016		2017		2018		2019	
	*B*	*P*	*B*	*P*	*B*	*P*	*B*	*P*	*B*	*P*	*B*	*P*
Gender	0.340	< 0.001	0.011	0.366	0.017	0.333	0.388	< 0.001	0.432	< 0.001	0.370	< 0.001
Time outdoors	0.140	0.056	0.940	< 0.001	0.964	< 0.001	0.113	0.147	−0.107	0.225	0.021	0.784
Bad habit 1	0.044	0.537	−0.012	0.717	−0.029	0.112	−0.050	0.526	0.135	0.139	0.112	0.145
Bad habit 2	−0.069	0.405	0.050	0.146	0.016	0.421	0.024	0.764	0.120	0.204	0.081	0.351
Bad habit 3	−0.084	0.246	0.000	0.985	−0.024	0.215	0.029	0.716	−0.008	0.930	−0.092	0.282
Bad habit 4	−0.043	0.594	−0.029	0.404	−0.024	0.220	−0.150	0.072	−0.025	0.794	0.012	0.891

Bad habit 1: Continuously using eyes for reading books, writing homework, or playing with electronic devices for 30–40 min; Bad habit 2: Excessive bending; Bad habit 3: Distance of less than 2 m when watching TV; Bad habit 4: Wrong sitting posture

**Table 4:** Multivariate linear regression analysis for potential factors associated with axial length in 5-year-old children

	2013		2014		2016		2017		2018		2019	
	*B*	*P*	*B*	*P*	*B*	*P*	*B*	*P*	*B*	*P*	*B*	*P*
Gender	0.348	< 0.001	−0.031	0.314	0.323	< 0.001	0.264	0.001	0.414	< 0.001	0.196	0.023
Time outdoors	−0.134	0.048	0.936	< 0.001	0.059	0.436	0.020	0.787	−0.183	0.018	−0.040	0.635
Bad habit 1	−0.015	0.822	0.018	0.589	0.179	0.018	−0.076	0.313	0.124	0.112	−0.023	0.794
Bad habit 2	−0.025	0.714	−0.013	0.713	−0.036	0.660	−0.006	0.936	−0.061	0.460	−0.015	0.873
Bad habit 3	−0.082	0.235	0.010	0.751	−0.024	0.773	0.055	0.470	−0.023	0.785	0.073	0.440
Bad habit 4	−0.112	0.110	0.031	0.390	0.060	0.437	−0.066	0.383	0.074	0.350	0.058	0.536

Bad habit 1: Continuously using eyes for reading books, writing homework, or playing with electronic devices for 30–40 min; Bad habit 2: Excessive bending; Bad habit 3: Distance of less than 2 m when watching TV; Bad habit 4: Wrong sitting posture

## Discussion

In the present study, the most common AL range was 22–23 mm and the most common refraction was emmetropia in 4- and 5-year-old children from 2013 to 2019, except for 2015. There were no significant increases of AL in 4- or 5-year-old children in Shanghai from 2013 to 2019. However, AL was mainly significantly associated with boys and time outdoors in the 4- and 5-year-old children, respectively. These results may be helpful for understanding the changing trend of AL and increasing prevalence of myopia in the younger generation of China.

In this study, the mean AL of 4- and 5-year-old children in Jinshan District was 22.21 ± 0.69 mm and 22.45 ± 0.70 mm, respectively, in agreement with previous studies carried out in Shanghai [8, 9]. Zhang et al. [8] found that the mean AL of 4- and 5-year-old children in Jiading and Xuhui District in 2013 was 22.18 ± 0.69 mm and 22.47 ± 0.71 mm, respectively, with significant difference between genders. He et al. [9] found that the mean AL of 4- and 5-year-old children in Jiading District in 2017 was 22.16 ± 0.65 mm and 22.32 ± 0.71 mm, respectively. Furthermore, the mean AL of 4–5-year-old children was reported to be 22.30 mm in Nanjing in 2016 [12], and 22.04 ± 0.68 mm in the USA [10]. In addition, the mean AL of 3–4-year-old children was 22.10 ± 0.79 mm in Pudong New District, Shanghai in 2017 [11], and the mean AL of 2–7-year-old children was 22.04 ± 0.92 mm in Korea from 1988 to 1989 [7]. The mean AL of 3-year-old children was calculated to be 22. 07 mm according to an established algorithm in Denmark in 1996 [16]. When comparing the AL among different studies, differences in age and AL measurement technique should be noted. Firstly, AL increases with age in children [10], suggesting that a wider age range will have an obvious effect on AL comparisons. Secondly, different AL measurement methods may also play an important role, such as IOL Master [8, 9, 11], an immersion A-scan sonogram [7, 10], or calculation [16]. In this study, the AL was measured using the IOL Master. The values obtained from the IOL Master would be larger than those obtained from an immersion A-scan sonogram theoretically, because the former measures the distance between the anterior surface of the tear film and the pigment epithelial without contact, whereas the ultrasonic measuring instrument measures the distance between the anterior surface of the cornea and the vitreous boundary membrane with contact. To the best of the authors’ knowledge, we first reported no significant AL changing trend over the last 7 years from 2013 to 2019. Lin et al. [17] found that the mean prevalence of myopia among 7-year-olds increased from 5.8% in 1983 to 21% in 2000. Holden et al. [18] estimated that the prevalence of myopia would increase from 23% in 2000 to 50% in 2050. We suggest that the prevalence of myopia in preschool children may not have increased from 2013 to 2019 due to the relative stability of AL. Rapid increases of AL and prevalence of myopia may occur in school-age children due to changes in social environmental risk factors such as aggravation of learning tasks, extension of learning time, additional after-school tutoring classes, etc.

In this study, parameters including horizontal corneal curvature, vertical corneal curvature, and height in both 4- and 5-year-old children and weight in 5-year-old children were significantly different from 2013 to 2019; however, few significant differences were observed in paired comparisons. Jiang et al. [19] found that flatter horizontal corneal curvature was significantly associated with larger corneal diameter in 4–18-year-old children, which was significantly associated with taller body height but not correlated with time outdoors. Furthermore, an abnormally large cornea (2.6%) and an abnormally small cornea (2.4%) [19] were not classified or interpreted in this study but may influence the mean value of corneal curvature. In addition, corneal curvature was also influenced by birth weight and gestational age [20–22]. Birth weight was negatively correlated with horizontal/vertical corneal curvature [20, 23]. Children’s height was significantly positively associated with boys, higher maternal height, higher maternal education levels, higher family income, and higher percentage of energy intake from protein [24]. Children’s weight was associated with eating rate and degree of chewing [25] and parental feeding practices [26]. This study did not collect and analyze these factors, but they may partly explain the differences between paired comparisons. Further study including these factors should be conducted.

Interestingly, our study found that AL was positively associated with time outdoors. Numerous studies have shown a protective effect of outdoor activities on myopia onset [27–31]. Addition of 40 min [27] or 80 min [31] of outdoor activity each day could reduce the incidence rate of myopia among primary-school-age children in China. Wu et al. [29] found that time outdoors of more than 11 h could lead to significantly less myopic shift and AL elongation. Deng et al. [32] found an overall protective effect against axial elongation from outdoor activities, albeit with a small and clinically nonsignificant overall treatment effect. The mechanism is generally accepted to be that sunlight-induced release of retinal dopamine could inhibit eyeball elongation [1, 33]. Therefore, besides time spent outdoors, outdoor lighting quality should also receive attention. This was just an observational study of AL in children from 2013 to 2019, and the relationship between AL elongation and outdoor activity time should be confirmed by longitudinal studies.

The present observational study suggested that AL was not associated with bad eyesight habits. Previous studies have shown that computer/Internet use was not related to incident myopia [34], and time and distance of near-work activity were not related to myopia progression [35], while protective behaviors related to close work (e.g., discontinuing close work every 30 min, longer distance in close work) could decrease myopia prevalence and reduce its progression [30]. But these studies did not measure AL. However, Ding et al. [36] found that longer near-work time was a risk factor for longer AL. Furthermore, Morgan et al. [37] suggested that intense schooling was linked to myopia, playing a major role in the current epidemic of myopia.

There may be some limitations to this study. Firstly, AL was analyzed in only one kindergarten with small sample size, which may not be representative of the whole of Shanghai. Secondly, refraction was determined by non-cycloplegic autorefraction, which may result in misclassification of refractive error, with an overestimation of myopia and underestimation of hyperopia. A difference of approximately 0.87 D was observed between cycloplegic and non-cycloplegic refraction among 4–6-year-old children in our previous study [38]. Thirdly, transfer and leaving of some students in this kindergarten may have some impact on the data analysis. For example, the sample size of 4-year-old children in 2013 should be the same as that of 5-year-old children in 2014, but they were different due to the transfer or departure of a number of children. Fourthly, this was not a complete observational study due to the lack of a visit in 2015. In addition, data on time outdoors and bad eyesight habits were collected using questionnaires based on self-reports by parents and guardians, which has limited validity. With the development of objective methods of measurement (e.g., FitSight fitness tracker [39] and light meters [29]), data collection will be more credible.

In conclusion, the AL of 4- and 5-year-old children remained relatively stable in Shanghai from 2013 to 2019, except for 2015. Longitudinal studies are needed to confirm the relationship between AL elongation and environmental risk factors.
